# The predictive value of Hemoglobin–Albumin–Lymphocyte–Platelet score for maximal cytoreduction surgery in advanced-stage epithelial ovarian cancer

**DOI:** 10.1590/1806-9282.20251837

**Published:** 2026-07-10

**Authors:** Gökçen Ege, Hande Nur Öncü, Neslihan Öztürk, Oğuz Kaan Köksal, Candost Hanedan, Vakkas Korkmaz

**Affiliations:** 1 Ankara Etlik City Hospital, Department of Gynecologic Oncology – Ankara, Turkey.

**Keywords:** Biomarkers, tumor, Cytoreduction surgical procedures, CA-125 antigen, Ovarian neoplasms, Risk assessment

## Abstract

**OBJECTIVE:**

The Hemoglobin–Albumin–Lymphocyte–Platelet score has shown prognostic value across various cancers; however, its utility in predicting cytoreductive outcomes in advanced epithelial ovarian cancer remains unclear. The aim of this study was to determine whether the preoperative Hemoglobin–Albumin–Lymphocyte–Platelet score predicts maximal cytoreduction at primary cytoreductive surgery.

**METHODS:**

In this single-center study, we analyzed data from 140 patients with epithelial ovarian cancer who underwent primary cytoreductive surgery at our clinic. Patients were categorized by residual disease: maximal cytoreduction (no macroscopic residual disease, n=104), optimal cytoreduction (n=19), and suboptimal cytoreduction (n=17). Preoperative Hemoglobin–Albumin–Lymphocyte–Platelet, CA-125, and clinical variables were compared across groups. Discrimination for maximal cytoreduction was assessed using receiver operating characteristic analysis.

**RESULTS:**

The maximal cytoreduction group had higher preoperative hemoglobin and lymphocyte counts, lower platelet counts, and lower CA-125 levels (p<0.005). Median Hemoglobin–Albumin–Lymphocyte–Platelet was significantly higher in maximal cytoreduction vs. optimal cytoreduction vs. suboptimal cytoreduction (35.37 vs. 21.10 vs. 20.19; all p<0.001). For predicting maximal cytoreduction, the Hemoglobin–Albumin–Lymphocyte–Platelet cutoff of 24.96 yielded a sensitivity of 71.4% and a specificity of 71.4% (area under the curve: 0.792; p<0.001). A CA-125 cutoff of 260.50 IU/mL provided a sensitivity of 71.4% and a specificity of 65.7% (area under the curve: 0.758; p<0.001).

**CONCLUSION:**

The preoperative Hemoglobin–Albumin–Lymphocyte–Platelet score demonstrates good discrimination for predicting maximal cytoreduction in advanced epithelial ovarian cancer and appears to perform at least comparably to CA-125. Given that patients selected for neoadjuvant chemotherapy were excluded, these performance estimates apply to a surgically selected population. Given its low cost and universal availability, Hemoglobin–Albumin–Lymphocyte–Platelet may support preoperative selection and counseling before cytoreductive surgery.

## INTRODUCTION

The Hemoglobin, Albumin, Lymphocyte, and Platelet (HALP) score was first described by Chen et al. in patients with gastric cancer^1^. It is calculated using the formula: hemoglobin×albumin×lymphocyte/platelet. Low hemoglobin levels contribute to intratumoral hypoxia and oxygen gradients, fostering phenotypic plasticity, genomic instability, and treatment resistance. Low albumin levels reflect malnutrition/inflammation and are correlated with postoperative complications and poorer survival after cytoreductive surgery (CR)^2^. Lymphocytes exert anti-tumor immunity, whereas thrombocytosis has been associated with tumor progression and poorer outcomes^3^. Moreover, platelets have been shown to play a role in tumor immunity, with elevated preoperative platelet levels associated with decreased survival^4^. Therefore, an elevated HALP score is generally expected to correlate with a better prognosis, whereas a reduced score is associated with poorer outcomes and diminished survival^1^.

The HALP score has been studied in various cancers, including gynecologic malignancies such as endometrial cancer^5^ and cervical cancer^6^. These studies have primarily focused on disease-free survival (DFS) and overall survival (OS). Indeed, meta-analyses have shown that a low HALP score is associated with decreased survival^7,8^. However, its significance in patients with ovarian cancer (OC) has not been explored to date.

In epithelial ovarian cancer (EOC), the most critical determinant of survival is maximal cytoreduction (MCR) in surgery, which means no visible residual tumor^9^. Achieving an MCR rate above 75% in OC surgery is associated with a 50% improvement in survival^10^. Therefore, identifying patients who are likely to achieve MCR preoperatively is a cornerstone of surgical management in OC. Various predictive models have been developed using radiological modalities, CA-125 levels, or laparoscopy; however, a universally accepted, standardized method has yet to be established. In light of this, we hypothesized that a higher preoperative HALP score is associated with achieving MCR at primary cytoreductive surgery (PCR). We therefore assessed the discriminatory performance of HALP, compared it with CA-125, and explored group-wise differences in routinely collected clinical and laboratory parameters. Importantly, HALP has not been evaluated in unselected EOC populations, and the discriminatory value in surgically selected cohorts may not reflect performance in broader clinical decision-making.

## METHODS

Following approval from the local ethics committee, all patients diagnosed with advanced-stage EOC and who underwent surgery at our clinic between October 2022 and April 2024 were consecutively included in this study. Our gynecologic oncology center has been operational for approximately 3 years; thus, the study window (October 2022–April 2024) reflects the full period of routine care available for case accrual at this institution. Patients with active infections, a history of another malignancy, recurrent disease, or incomplete records were excluded. Because this study aimed to evaluate HALP as a pre-treatment predictor in patients undergoing PCR, we excluded patients triaged to neoadjuvant chemotherapy (NACT) based on initial biopsy findings, as well as those who had already received NACT and were later referred for interval debulking. NACT substantially alters hematologic and inflammatory markers, making the HALP score no longer representative of the patient's actual baseline status. Including such patients would have compromised the biological validity of HALP and introduced a different type of bias.

All surgeries were performed by gynecologic oncologists, and the pathological specimens were analyzed by gynecopathologists. Surgical treatment included total abdominal hysterectomy, bilateral salpingo-oophorectomy, metastasectomy, or total/partial resection of the involved organ in cases of extensive disease and pelvic and para-aortic lymph node dissection when necessary. Based on surgical outcomes, patients with no macroscopic residual tumor were classified as MCR (Group 1), those with residual tumors less than 1 cm as optimal cytoreduction (OCR) (Group 2), and those with residual tumors larger than 1 cm as suboptimal cytoreduction (SCR) (Group 3). The SCR subgroup included patients in whom cytoreduction was abandoned at the beginning of the procedure due to clear intraoperative irresectability. These were effectively "inoperable" cases, biologically corresponding to the group often triaged to NACT in routine practice.

Demographic data, including patient age, body mass index (BMI), comorbidities, smoking status, Eastern Cooperative Oncology Group (ECOG) performance status, and American Society of Anesthesiologists (ASA) performance scores, were recorded. Preoperative blood tests, including complete blood counts, serum biochemistry, and CA-125 levels, obtained within 14 days before surgery, were used to calculate HALP. Hemoglobin (Hb), albumin (Alb), lymphocyte (Ly), and platelet (Plt) values were documented. The HALP score was calculated as (Hb [g/L]×albumin [g/L]×lymphocyte count [/L])/platelet count [/L]^6,11^. For laboratories reporting Hb/Alb in g/dL and counts in 10³/μL, the numerical value on this g/L,/L scale equals 100×(Hb[g/dL]×Alb[g/dL]×lymphocyte[10³/μL]/platelet[10³/μL]) [for example, Hb 12.0 g/dL, Alb 4.0 g/dL, lymphocyte 2.0×10³/μL, platelets 300×10³/μL → HALP=100×(12×4×2/300)=32.0].

Patient records were meticulously documented, including surgical notes, outcomes, intraoperative and postoperative complications, pathology results, and recovery details. The type and duration of the operation, FIGO 2014 stage^12^, tumor histological subtype, and the amount of ascites were also recorded. Ascites volume was categorized into three groups based on intraoperative measurement:

- Minimal/none: <200 mL
- Moderate: 200–1,000 mL
- Massive: >1,000 mL

This categorization was used for both univariate comparisons and multivariable logistic regression.

### Statistical analysis

Analyses were performed in SPSS v21 (IBM Corp.). Continuous variables were summarized as median (range) or mean±SD, and categorical variables as n (%). Because CA-125 values showed a right-skewed distribution, they were log-transformed (ln[CA-125]) for regression analyses to improve model fit and stabilize variance. Normality was assessed using the Shapiro-Wilk test and Q–Q plots. Between-group comparisons used analysis of variance (ANOVA) (parametric) or Kruskal-Wallis (non-parametric) for three groups with Bonferroni- or Dunn-adjusted pairwise tests as appropriate; two-group comparisons used Student's t-test or Mann-Whitney U. Categorical variables were compared using the χ^2^ or Fisher's exact test. Discrimination for the primary endpoint (MCR vs. non-MCR) was evaluated using receiver operating characteristic (ROC) analysis with area under the curve (AUC) for HALP and CA-125; optimal cutoffs were derived by Youden's index. AUCs were compared using DeLong's test. Where available, 95%CIs were estimated. A two-sided p<0.05 was considered statistically significant.

### Primary endpoint

Our primary endpoint was MCR at the time of PCR. For analysis, we grouped OCR and SCR as "non-MCR."

### Multivariable analysis

A multivariable logistic regression model was constructed, including HALP score, ln-transformed CA-125, and ascites volume, to assess independent predictive value. Categorical variables were dummy-coded, and model fit was evaluated with the Hosmer-Lemeshow test.

## RESULTS

**Figure 1:**
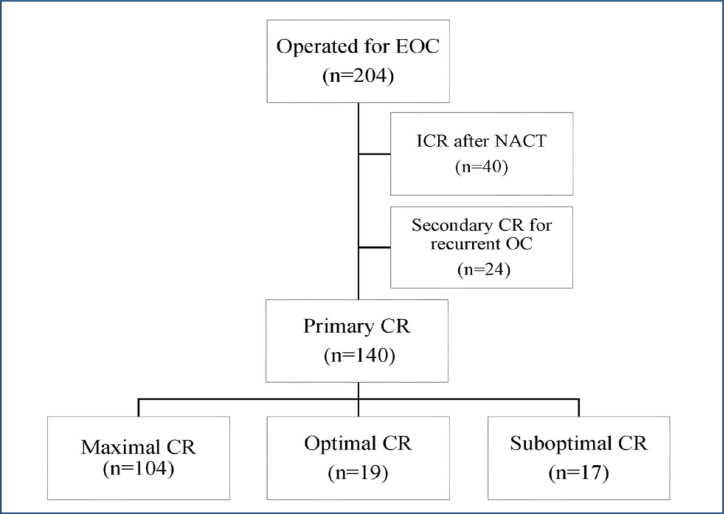
Flowchart of patient selection. CR: cytoreduction; EOC: epithelial ovarian cancer; IDS: interval cytoreduction; NACT: neoadjuvant chemotherapy; OC: ovarian cancer.

During the study period, a total of 204 patients with advanced-stage EOC underwent surgery at our clinic. Of these, 24 patients were excluded due to disease recurrence, and 40 were excluded after being selected for NACT. Ultimately, 140 patients who underwent PCR were included in the analysis. Among these, 104 patients (74.2%) achieved MCR, 19 patients (13.5%) achieved OCR, and 17 patients (12.1%) had SCR (Figure 1).

### Comparisons between groups

Median age was 57.5 years (19–79) in MCR, 59 (31–85) in OCR, and 59 (20–80) in SCR (p=0.439). BMI was 29 (17–47) kg/m^2^ in MCR, 27 (18–31) in OCR, and 30 (18–39) in SCR, with a significant difference between MCR and OCR (p=0.016), although this may not remain significant after correction for multiple comparisons.

Preoperative hemoglobin (Hb) was higher in MCR than in OCR and SCR (12.9 [9.1–15.8] vs. 11.3 [9.6–13.4] vs. 11.5 [8.7–13.4] g/dL; p<0.001). Lymphocyte counts were higher in MCR than SCR (2.01 [1.02–4.14] vs. 1.58 [0.92–2.84] 10³/μL; p=0.005), with no significant differences in other pairwise comparisons. Platelet counts were lower in MCR than in OCR and SCR (325 [160–977] vs. 422 [268–870] vs. 408 [227–657] 10³/μL; p<0.001).

Operative time differed significantly across groups (OCR: 360 [120–540] min, MCR: 245 [90–480] min, SCR: 110 [60–210] min; p<0.001). The short operative times observed in the SCR group likely reflect early intraoperative assessment leading to abortion of cytoreduction rather than completion of a full debulking attempt.

Intraoperative ascitic fluid volume was significantly lower in MCR than in OCR and SCR (median 100 [50–8,000] vs. 1,000 [50–7,000] vs. 3,000 [200–9,000] mL; p<0.001).

Preoperative CA-125 was lower in MCR than in OCR and SCR (143 [9–70,818] vs. 508 [118–5,782] vs. 1,233 [32–9,427] IU/mL; p<0.001). HALP was higher in MCR than in OCR and SCR (35.37 [8.32–86.90] vs. 21.10 [7.34–53.57] vs. 20.19 [6.37–34.61]; p<0.001). Baseline demographics and preoperative laboratory values are summarized in Table 1.

**Table 1:** Comparison of patients’ demographic and biochemical parameters.

		Group 1	Group 2	Group 3	p-value
Age (years) Median (min–max)		57.5 (19–79)	59 (31–85)	59 (20–80)	0.439
BMI (kg/m^2^) Median (min–max)		29 (17–47)	27 (18–31)*	30 (18–39)	0.016
Smoking n, (%)		17 (16.3%)	4 (21%)	1 (5.8%)	0.432
DM n, (%)		25 (24%)	6 (31.5%)	6 (35.3%)	0.535
HT n, (%)		32 (30.7%)	6 (31.5%)	9 (52.9%)	0.196
Pulmonary disease (including COPD) n, (%)		5 (4.8%)	3 (15.7%)	2 (11.7%)	0.170
CAD n, (%)		12 (11.5%)	1 (5.2%)	2 (11.7%)	0.710
ASA score n, (%)					
	I	3 (2.8%)	–	–	0.531
	II	74 (71.1%)	11 (57.8%)	11 (64.7%)	
	III	27 (25.9%)	8 (42.1%)	6 (35.3%)	
ECOG score n, (%)					
	0	42 (40.3%)	4 (21%)	5 (29.4%)	0.303
	1	58 (55.7%)	13 (68.4%)	10 (58.8%)	
	2	4 (3.8%)	2 (10.5%)	2 (11.7%)	
Preoperative Hb (g/dL) Median (min–max)		12.9 (9.1–15.8)	11.3 (9.6–13.4)*	11.5 (8.7–13.4)*	<0.001
Lymphocytes (10^3^/μL) Median (min–max)		2.01 (1.02–4.14)	1.64 (1.18–6.53)	1.58 (0.92–2.84)*	0.005
Platelets (10^3^/μL) Median (min–max)		325 (160–977)	422 (268–870)*	408 (227–657)*	<0.001
Preoperative Alb (g/dL) Median (min–max)		4.1 (2.4–4.9)	3.9 (2.6–4.6)	3.9 (2.7–4.6)	0.038
Preoperative CA-125 Median (min–max)		143.5 (9–70,818)	508 (118–5,782)*	1,233 (32–9,427)*	<0.001
Intraoperative ascites (cc) Median (min–max)		100 (50–8,000)	1,000 (50–7,000)*	3,000 (200–9,000)*	<0.001
Operation time (minutes) Median (min–max)		245 (90–480)	360 (120–540)*	110 (60–210)*, **	<0.001
HALP score Median (min–max)		35.37 (8.32–86.90)	21.10 (7.34–53.57)*	20.19 (6.37–34.61)*	<0.001

* p<0.05: vs. Group 1.

** p<0.005: vs. Group 2. BMI: body mass index; ASA: American Society of Anesthesiologists; ECOG: Eastern Cooperative Oncology Group; HALP: Hemoglobin–Albumin–Lymphocyte–Platelet; DM: diabetes mellitus; HT: hypertension; COPD: chronic obstructive pulmonary disease; CAD: coronary artery disease; CA-125: cancer antigen 125; Hb: hemoglobin; Alb: albumin.

The most common histopathological subtype was high-grade serous carcinoma (71.4%), followed by clear cell carcinoma (8.6%), endometrioid carcinoma (7.8%), high-grade mucinous carcinoma (7.1%), low-grade serous carcinoma (4.3%), and Brenner tumor (0.7%).

### Receiver operating characteristic analysis

**Figure 2:**
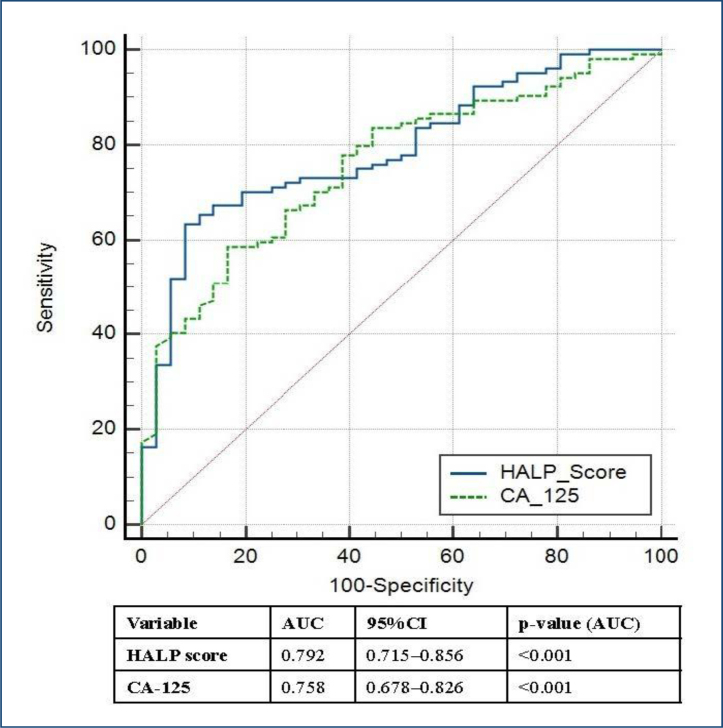
Receiver operating characteristic curves comparing the Hemoglobin–Albumin–Lymphocyte–Platelet score and CA-125 for predicting maximal cytoreduction in advanced epithelial ovarian cancer. AUC: area under the curve; 95%CI: 95% confidence interval.

ROC analysis identified an optimal HALP cutoff of 24.96 for predicting MCR (AUC=0.792, sensitivity 71.4%, specificity 71.4%; p<0.001) and a CA-125 cutoff of 260.50 IU/mL (AUC=0.758, sensitivity 71.4%, specificity 65.7%; p<0.001). Comparison of the two ROC curves using the DeLong test revealed no significant difference between HALP and CA-125 (ΔAUC=0.034, p=0.53), indicating that HALP performs comparably to CA-125 in discriminating MCR from non-MCR. ROC curves comparing HALP and CA-125 with AUC values and DeLong p-value are presented in Figure 2.

### Multivariate logistic regression

In multivariable logistic regression including HALP, ln-CA125, and ascites category, the HALP score remained an independent predictor of achieving MCR (OR 0.948, 95%CI 0.911–0.987, p=0.009). ln(CA-125) was not independently associated with surgical outcome (p=0.276). Moderate ascites significantly reduced the likelihood of MCR (OR 0.177, p=0.018), while massive ascites showed a non-significant trend toward lower MCR rates (p=0.092).

## DISCUSSION

In this cohort of 140 patients with advanced-stage EOC undergoing CR, both preoperative CA-125 and HALP were significant predictors of MCR. The HALP score showed sensitivity (71.4%) and specificity (71.4%) at least comparable to those reported for laparoscopic scoring approaches.

CA-125 is a well-established biomarker in EOC, and its predictive value has been extensively studied. A 2010 meta-analysis reported that lower CA-125 levels were generally associated with successful cytoreduction; however, variability in cytoreduction rates and the absence of a standardized threshold have limited the adoption of a universal cutoff^13^. In a 2004 study, Eltabbakh et al. identified CA-125 ≤500 IU/mL as a valuable predictor of complete cytoreduction (sensitivity 73%, specificity 74%), with 31.9% achieving complete and 48.6% OCR^14^. In 2009, Vorgias et al. reported that a 500 IU/mL threshold predicted OCR with 78.5% sensitivity and 89.6% specificity in stage III–IV disease^15^. However, that study addressed optimal rather than MCR. In our cohort, a CA-125 cutoff of 260.5 IU/mL predicted MCR with 71.4% sensitivity and 65.7% specificity. Sensitivities and specificities near 72% are insufficient for standalone triage and may only support, but not replace, clinical and radiologic assessment.

Beyond serum markers, laparoscopic assessment of resectability has been widely studied. In 2006, Fagotti et al. developed a laparoscopic scoring system to predict cytoreductive success, which involved the laparoscopic assessment and scoring of all abdominal organs for tumor metastasis. A threshold score of ≥8 predicted cytoreduction with 30% sensitivity, 100% specificity, and 74% accuracy^16^. However, this study was designed to predict OCR. Petrillo et al. later assessed the predictive value of the Fagotti score for MCR in a 2015 study, finding that a score of ≥10 indicated a 0% likelihood of MCR, a 33.2% risk of unnecessary laparotomy, and an AUC of 0.885^17^. While informative, laparoscopy entails additional surgical and anesthetic exposure, particularly relevant for patients with limited fitness, and does not eliminate the risk of non-therapeutic laparotomy. These comparisons should be interpreted cautiously, as laparoscopic scores directly assess tumor burden, whereas HALP reflects systemic inflammatory and nutritional status.

The HALP score, initially developed for non-gynecologic cancers, has also shown promise as a predictive tool in gynecologic malignancies. Previous studies have reported median cutoffs of approximately 31.2 for non-gynecologic cancers and 23.1 for gynecological cancers. Leetanaporn et al. found that a HALP score of 22.2 or higher was associated with improved progression-free survival and OS in locally advanced cervical cancer (LACC)^6^. In endometrial cancer, a cutoff near 24 correlated with adverse pathologic features, although survival associations were inconsistent^5^. Consistent with these data, we observed that a HALP cutoff of 24.96, derived via Youden's index, predicted MCR with 71.4% sensitivity and 71.4% specificity—at least comparable to CA-125 and consistent with values reported for laparoscopic scoring systems.

Although HALP showed slightly higher discriminative ability compared with CA-125, the difference between the two AUCs was not statistically significant in the DeLong analysis. Therefore, HALP should not be interpreted as superior to CA-125, but rather as a complementary biomarker that may strengthen preoperative risk stratification when used alongside established clinical and radiologic parameters. In the multivariable model, HALP but not ln(CA-125) remained independently associated with MCR, suggesting that HALP captures biological domains (inflammation and nutritional status) that are not fully reflected by tumor burden markers alone.

Limitations include the retrospective single-center design, a modest sample size, and a lack of long-term follow-up. In addition, our hospital has been in operation for only 3 years, which constrained the sample size and precluded mature DFS/OS follow-up within the study window. Another major limitation of this study is that patients selected for NACT—either at initial presentation or following treatment elsewhere—were excluded. This resulted in a surgically favorable cohort, likely overestimating HALP's predictive performance. Including NACT-treated patients would have introduced a different type of bias, as chemotherapy profoundly alters serum albumin, lymphocyte, and platelet counts and would dilute the discriminatory ability of HALP. Similarly, most patients categorized as SCR were cases in whom irresectability was evident at the beginning of surgery, effectively mirroring the clinical profile of patients typically triaged to NACT. This explains the short operative times observed in the SCR group and clarifies the biological distinction between MCR-achievable and non-MCR cohorts.

Strengths include the high MCR rate, consistent with contemporary series, and the reliance on objective preoperative measures readily obtainable in routine practice.

In conclusion, HALP is a simple, low-cost, and objective adjunct for preoperative triage toward MCR in advanced EOC. Given the exclusion of NACT-eligible patients, these findings reflect performance within a surgically selected cohort. HALP may provide additional support in preoperative triage but requires validation in broader, unselected populations before clinical adoption.

## Data Availability

De-identified source data and SPSS syntax have been deposited on Zenodo (CC BY 4.0): DOI: 10.5281/zenodo.17329833. The deposit includes a CSV dataset, a data dictionary, and SPSS syntax sufficient to reproduce the summary tables and ROC analyses. The datasets generated and/or analyzed during the current study are available from the corresponding author upon reasonable request.
